# Hair product use, age at menarche and mammographic breast density in multiethnic urban women

**DOI:** 10.1186/s12940-017-0345-y

**Published:** 2018-01-04

**Authors:** Jasmine A. McDonald, Parisa Tehranifar, Julie D. Flom, Mary Beth Terry, Tamarra James-Todd

**Affiliations:** 1 Department of Epidemiology, Mailman School of Public Health, Columbia University Medical Center, 722 West 168th Street, New York, NY 10032 USA; 2 Herbert Irving Comprehensive Cancer Center, Columbia University Medical Center, New York, NY 10032 USA; 3 Departments of Environmental Health and Epidemiology, Harvard T.H. Chan School of Public Health, 665 Huntington Ave., Bldg. 1, 14th Floor, Boston, MA 02115 USA; 4 Division of Women’s Health, Department of Medicine, Brigham and Women’s Hospital, Boston, MA 02115 USA

**Keywords:** Menarche, Mammographic breast density, Endocrine disrupting chemicals, Cosmetics

## Abstract

**Background:**

Select hair products contain endocrine disrupting chemicals (EDCs) that may affect breast cancer risk. We hypothesize that, if EDCs are related to breast cancer risk, then they may also affect two important breast cancer risk factors: age at menarche and mammographic breast density.

**Methods:**

In two urban female cohorts (*N* = 248): 1) the New York site of the *National Collaborative Perinatal Project* and 2) the *New York City Multiethnic Breast Cancer Project*, we measured childhood and adult use of hair oils, lotions, leave-in conditioners, root stimulators, perms/relaxers, and hair dyes using the same validated questionnaire. We used multivariable relative risk regression models to examine the association between childhood hair product use and early age at menarche (defined as <11 years of age) and multivariable linear regression models to examine the association between childhood and adult hair product use and adult mammographic breast density.

**Results:**

Early menarche was associated with ever use of childhood hair products (RR 2.3, 95% CI 1.1, 4.8) and hair oil use (RR 2.5, 95% CI 1.2, 5.2); however, additional adjustment for race/ethnicity, attenuated associations (hair products RR 1.8, 95% CI 0.8, 4.1; hair oil use RR 2.3, 95% CI 1.0, 5.5). Breast density was not associated with adult or childhood hair product or hair oil use.

**Conclusions:**

If confirmed in larger prospective studies, these data suggest that exposure to EDCs through hair products in early life may affect breast cancer risk by altering timing of menarche, and may operate through a mechanism distinct from breast density.

## Background

Recent trends suggest a convergence in the incidence of breast cancer rates in black and white women due to the stability in incidence trends in white women compared to the steady annual increase in black women (0.3% per year) [1, 2]. These changing incidence patterns over a short time period cannot be attributed to changing genetic factors. As such, it is important to consider the role of environmental exposures that may differ in black and white women. Given that breast cancer risk may be most influenced when the breast changes in structure and function, consideration of environmental exposures during critical and sensitive windows of susceptibility is essential for providing evidence on new risk factors.

Endocrine disrupting chemicals (EDCs) have been posited as potential environmental factors that could affect breast cancer risk through their ability to have direct action on hormone receptors; interference with transport or feedback; behave as agonist or antagonist of nuclear receptors; or confer epigenetic changes that increase oncogenic susceptibility [3–5]. Of particular concern, EDCs may be more deleterious when the breast tissue is highly proliferative, such as during puberty [6]. Clinical, epidemiological, and laboratory studies have suggested EDCs are associated with earlier pubertal events [6] and timing of pubertal maturation is associated with breast cancer risk [7]. For every 1-year delay in menarche, the risk of breast cancer decreases by around 5% [8, 9]. Further, certain EDCs are important breast carcinogens that may impact breast tissue remodeling, which in turn may affect mammographic breast density [10], a measure of the amount of fibroglandular breast tissue and one of the strongest risk factors for breast cancer [11–13].

Hair products that contain estrogens or xenoestrogens may interfere with the normal activity of the endocrine system through hormonal dysregulation [4]. Natural and synthetic EDCs are added to hair products to encourage hair growth (estrogen), provide scent (phthalates), and for product preservation (parabens) [3, 14]. Women and certain racial/ethnic minorities tend to be frequent and long-term users of such products, which are applied directly to the hair and scalp [3, 15, 16]. The *Black Women’s Health Study* surveyed African American women and found that 94% under age 45 and 89% over age 45 use perms or relaxers [17]. The *Women’s Circle of Health Study* corroborates findings, reporting 87% of African American women, but 6% of European American women have a history of regularly using chemical relaxers [18]. Moreover, in the *Greater New York Hair Products Study*, of 16 specific brands of hair products that were identified as commonly used among the participants, 69% of the products contained parabens [15]. This routine and chronic use of products containing EDCs may impact breast cancer; however, few studies have evaluated hair product use and breast cancer risk factors [19, 20].

To address this gap, we examine the association of hair product use with two important breast cancer risk factors - age at menarche and mammographic breast density. We hypothesize that the use of hair products in childhood is associated with earlier age at menarche and hair product use in childhood and adulthood may be associated with greater mammographic breast density.

## Methods

We used data from an adult follow-up study of the New York site of the *National Collaborative Perinatal Project* (hereafter referred to as NY-NCPP) that included women born between 1959 and 1963 and followed up between 2001 and 2006 (for details see [21–24]). We also used data from the *New York City Multiethnic Breast Cancer Project* (hereafter referred to as NY-MBCP), which recruited women between the ages of 40 and 60 years from 2007 to 2008 (for details see [25, 26]). The two studies followed similar protocols and used the same validated questions on hair product use and also the same methods for assessing mammographic breast density. We collected information on hair product use and mammograms from subsets of these cohorts; the final combined sample included 248 women (98 from NY-NCPP and 150 from NY-MBCP). The Internal Review Boards at Columbia University, Long Island University, and Long Island College Hospital in Brooklyn approved these studies.

Study participants were asked to complete questions on hair product use in childhood (before age 13) and adulthood (since age 20); specifically ever use of hair oils, lotions, leave-in conditioners, root stimulators, perms/relaxers, and hair dyes as previously described [19]. Hair product use was defined in three ways for each time period: 1) ever use of *any* category of hair product (referred to as ever use), 2) ever use of hair oils (referred to as hair oils), 3) ever use of any other category of hair care product (excluding hair oils; referred to as other products) [15, 19]. For the latter category, we collapsed hair lotions, leave-in conditioners, root stimulators, perms/relaxers, and hair dyes into this category due to too few participants reporting use [19]. We defined the childhood *ever use* exposure variables as ever using hair products before age 13. We calculated the duration of childhood use of hair oils based on the assumption that, once a woman reported the use of hair oils, she would continue to use them until age 13. Therefore, we calculated duration based on the age women reported they started using hair oils up until age 13. For women who reported their age at menarche occurring before age 11, their childhood hair product exposure occurred prior to menarche. In other words, among women who reported experiencing menarche before age 11, all had hair product use before age 11. We defined adult *ever use* exposure variables as ever using hair products at or after age 20. We calculated the duration of adult use of hair oils as the difference between the time of first use at age 20 or older and the woman’s age at interview or when she reported stopping product use.

We examined two breast cancer risk factors: age at menarche and mammographic breast density. Age at menarche was self-reported by participants (for details see [23, 25]). In both studies, mammograms were only requested if the woman already received a screening mammogram. For NY-NCPP, among all available mammograms, we used the mammogram taken closest to the interview data collection. The majority of mammograms for the NY-MBCP projects were obtained on the same day as the interviews that collected hair product use and other covariate data. Details on mammographic density assessment have been described previously for NY-NCPP [24] and for NY-MBCP [25]. We excluded women who had a history of breast cancer. Briefly, we measured dense breast area and total breast area (measured in pixels and converted to cm^2^), and calculated percent density as total dense area divided by total breast area, multiplied by 100. Pearson correlation of reader reliability for both studies ranged from 0.87–0.99 for breast area and 0.90–0.93 for dense area.

For covariates, we collected sociodemographic data (age, education, and race/ethnicity) and health information (self-reported height and weight) through a questionnaire. Race/ethnicity was based on adult self-report as described previously [25–27]. We categorized race and ethnicity as Hispanic, non-Hispanic black, and non-Hispanic white. For our analyses, we included women who had complete information on hair product use in childhood and adulthood (*n* = 98 from NY-NCPP and *n* = 150 from NY-MBCP).

After descriptive analyses, we used multivariable relative risk regression using the binomial link to examine the association between childhood hair product use and the age at menarche (<11 years compared to ≥11 years (referent)). We also conducted a sensitivity analysis modeling age at menarche as a continuous variable using linear regression. Additionally, we conducted sensitivity analyses to determine if the association between early life exposure of hair product use and age at menarche had a stronger effect in younger women (independently defined as under age 50 or not postmenopausal). We also used logistic regression to model duration of childhood hair oil use and the age at menarche when adjusted for age at interview and site only. Additionally, we adjusted for race/ethnicity. However, the additional adjustment for race/ethnicity could lead to structural confounding due to minimal overlap between racial/ethnic groups with respect to hair product use. As such, models adjusted for race/ethnicity should be interpreted with caution. We assessed age at interview, cohort, and race/ethnicity as potential confounders and included them in the final model, if they altered the association between childhood hair product use and age at menarche by more than 10% in the multivariable models. To determine the association between hair product use and mammographic breast density (percent density and total dense area), we performed linear regression. We log transformed the mammographic breast density measures for the regression analyses. We also modeled duration of adult hair oil use and mammographic breast density measures when adjusted for age at interview, BMI, site, and race/ethnicity. We assessed age at interview, cohort, adult BMI, and race/ethnicity as potential confounders and included them in the model if they altered the association between adult hair product use and mammographic breast density measures by more than 10% in the multivariable models. Again, structural confounding for models adjusted for race/ethnicity could be a potential issue and should be interpreted with caution. As such, models with and without adjustment for race/ethnicity are presented. For all analyses, we modeled exposure of hair product use and duration as defined above. We performed all analyses in the combined sample of both cohorts, and stratified by cohort membership.

## Results

Table 1 details the differences between epidemiologic factors, breast cancer risk factors, and the use of hair products by cohort. We found that childhood ever use of any hair product and childhood ever use of hair oil were associated with a higher probability of reaching menarche before 11 years of age (Table 2). We also observed a non-significant, positive association between menarche before age 11 and ever use of other hair products in childhood (Table 2). After adjusting for race/ethnicity, the associations were directionally similar, though non-significant (childhood any hair product use RR 1.8, 95% CI 0.8, 4.1; hair oil RR 2.3, 95% CI 1.0, 5.5; and other products RR 1.5, 95% CI 0.7, 3.2). When stratified by race/ethnicity, we found the association between childhood hair oil ever use and menarche were directionally similar, though non-significant, in Hispanic (RR 2.91, 95% CI 0.9, 9.2) and non-Hispanic black women (RR 1.8, 95% CI 0.6, 5.3). There were no non-Hispanic white women that reported using hair products before age 11 years. Given the categorical outcome, we had low power to look at associations by the cohorts separately. However, we did observe cohort-specific associations between childhood hair product use and age at menarche before 11 years of age were in the same direction as our overall findings (data not shown).

**Table 1:** Distribution of epidemiologic factors, breast cancer risk factors, and hair product use in the New York National Collaborative Perinatal Project (NY-NCPP) and the New York City Multiethnic Breast Cancer Project (NY-MBCP)

	Overall (*N* = 248)	NY-NCPP (N = 98)	NY-MBCP (N = 150)
	Mean ± SD or N (%)	Mean ± SD or N (%)	Mean ± SD or N (%)
*Sociodemographic factors*			
Age (years)	46.8 ± 5.9	42.0 ± 1.8	49.9 ± 5.5*
Race/Ethnicity			
Hispanic	60 (24.2)	37 (37.8)	23 (15.3)
non-Hispanic black	123 (49.6)	33 (33.7)	90 (60.0)*
non-Hispanic white	65 (26.2)	28 (28.6)	37 (24.7)
Education			
High school or less	52 (21.1)	11 (11.2)	41 (27.5)*
Some college	84 (34.0)	37 (37.8)	47 (31.5)
College or more	11 (44.9)	50 (51.0)	61 (40.9)
*Breast Cancer Risk Factors*			
Body Mass Index (kg/m^2^)	29.0 ± 6.9	27.8 ± 6.8	29.8 ± 6.9*
Body Mass Index (kg/m^2^)			
< 25	77 (32.1)	37 (39.4)	40 (27.4)
≥ 25	163 (67.9)	57 (60.6)	106 (72.6)
Breast Percent Density (%)	15.4 ± 12.5	18.9 ± 13.3	13.1 ± 11.4*
Breast Dense Area (cm^2^)	20.0 ± 16.1	23.7 ± 17.9	17.5 ± 14.4*
Age at menarche	12.4 ± 1.7	12.5 ± 1.6	12.3 ± 1.7
Age at menarche			
< 11 years	26 (11.3)	7 (8.9)	19 (12.7)
≥11 years	203 (88.7)	72 (91.1)	131 (87.3)
Menopausal status			
Pre/Peri-menopausal	176 (75.5)	79 (95.2)	97 (64.7)*
Postmenopausal	57 (24.5)	4 (4.8)	53 (35.3)
*Childhood Use of Hair Products (ever use)*	106 (42.7)	35 (35.7)	71 (47.3)
Hair oils	92 (37.1)	29 (29.6)	63 (42.0)
Hair lotions	24 (9.7)	13 (13.4)	11 (7.3)*
Root stimulators	3 (1.2)	–	3 (2.0)
Leave-in conditioners	20 (8.1)	12 (12.2)	8 (5.3)
Perms/relaxers	15 (6.1)	9 (9.2)	6 (4.0)
Hair dyes	2 (0.8)	–	2 (1.3)
Overall duration of childhood use of hair oil (years)	7.8 ± 3.1	7.7 ± 3.1	7.8 ± 3.2
*Adult Use of Hair Products (ever use)*	229 (92.3)	85 (86.7)	144 (96.0)*
Hair oils	117 (47.2)	33 (33.7)	84 (56.0)*
Hair lotions	109 (44.0)	31 (31.6)	78 (52.0)*
Root stimulators	38 (15.3)	9 (9.2)	29 (19.3)*
Leave-in conditioners	140 (56.5)	54 (55.1)	86 (57.3)
Perms/relaxers	153 (61.7)	53 (54.1)	100 (66.7)*
Hair dyes	153 (61.7)	60 (61.2)	93 (62.0)
Overall duration of adult use of hair oil (years)	25.4 ± 7.1	21.4 ± 3.3	26.9 ± 7.7*

*p < 0.05; represents the test for differences between cohorts

**Table 2:** Binomial regression of childhood hair product use and age at menarche (<11 vs. ≥11 (referent) years) in the New York National Collaborative Perinatal Project (NY-NCPP) and the New York City Multiethnic Breast Cancer Project (NY-MBCP)

	*n* < 11/≥11	Age and cohort adjusted^a^	Age, cohort, and race/ethnicity adjusted^b^
		RR (95% CI)	RR (95% CI)
Childhood use			
Never use	10/125	1.00	1.00
Ever use	16/78	2.26 (1.05, 4.85)	1.79 (0.79, 4.07)
Hair oils			
Never use	11/137	1.00	1.00
Ever use	15/66	2.47 (1.16, 5.25)	2.32 (0.98, 5.48)
Other products			
Never use	19/169	1.00	1.00
Ever use	7/33	1.78 (0.80, 3.94)	1.45 (0.65, 3.22)

The model is adjusted for age at interview^a, b^ (continuous), cohort^a, b^ (NY-NCPP or NY-MBCP), and race ethnicity ^b^ (Hispanic, non-Hispanic black, non-Hispanic white)

In the sensitivity analysis of hair product use and age at menarche, where menarche was modeled continuously, we observed a non-significant inverse relationship, suggesting childhood hair product use was associated with earlier age at menarche (childhood any hair product use β -0.3, 95% CI -0.8, 0.1; hair oil β -0.2, 95% CI -0.7, 0.2; and other products β -0.3, 95% CI -0.9, 0.2). In an additional sensitivity analysis where we adjusted for age at interview and cohort, associations between childhood hair product use (ever use, hair oil use, and other use) and categorical age at menarche (i.e. before age 11 v. at or after age 11 years) were stronger when we limited analyses to women under 50 years of age (all models *P < 0.05*) or restricted to those who were not postmenopausal (all models *P < 0.05*). In these latter sensitivity analyses, sample size restricted our ability to adjust for race/ethnicity, as only non-Hispanic black women remained in the models when restricting to younger women (data not shown).

There was no association between childhood hair product use and mammographic breast density measures (Table 3). There was a statistically significant association between other adult hair product use and mammographic percent density and dense area after adjusting for age at interview, BMI, and cohort (Table 3). Estimates did not change with additional adjustment for race/ethnicity (data not shown). Upon examination of individual hair products, adult hair dye use was associated with a slight increase in percent mammographic density (β 0.4, 95% CI 0.1, 0.6) and dense area (β 0.3, 95% CI 0.03, 0.5). Findings were similar when limited to women under age 50.

**Table 3:** Linear regression of childhood and adult hair product use and mammographic breast density in the New York National Collaborative Perinatal Project (NY-NCPP) and the New York City Multiethnic Breast Cancer Project (NY-MBCP)

	Overall^a^	NY-NCPP^b^	NY-MBCP^b^
	β (95% CI)	β (95% CI)	β (95% CI)
*Percent Density*			
Childhood use	0.004 (−0.23, 0.24)	0.12 (−0.25, 0.49)	−0.08 (−0.38, 0.22)
Hair oils	−0.04 (−0.28, 0.20)	0.07 (−0.33, 0.46)	−0.14 (−0.44, 0.16)
Other Products	0.07 (−0.23, 0.36)	0.13 (−0.28, 0.54)	−0.06 (−0.47, 0.35)
*Dense Area*			
Childhood use	0.03 (−0.19, 0.26)	0.15 (−0.21, 0.51)	−0.05 (−0.33, 0.23)
Hair oils	−0.04 (−0.27, 0.19)	0.07 (−0.31, 0.45)	−0.13 (−0.41, 0.16)
Other Products	0.09 (−0.18, 0.37)	0.16 (−0.23, 0.55)	−0.02 (−0.41, 0.37)
	β (95% CI)	β (95% CI)	β (95% CI)
*Percent Density*			
Adult use	0.34 (−0.09, 0.77)	0.44 (−0.07, 0.95)	0.03 (−0.70, 0.76)
Hair oils	0.08 (−0.15, 0.32)	0.02 (−0.37, 0.41)	0.03 (−0.27, 0.33)
Other Products	0.41 (0.02, 0.79)	0.49 (−0.001, 0.98)	0.22 (−0.38, 0.82)
*Dense Area*			
Adult use	0.35 (−0.06, 0.76)	0.45 (−0.05, 0.95)	0.06 (−0.64, 0.75)
Hair oils	0.06 (−0.16, 0.28)	−0.10 (−0.48, 0.27)	0.08 (−0.20, 0.36)
Other Products	0.41 (0.04, 0.78)	0.55 (0.07, 1.02)	0.17 (−0.41, 0.74)

All models are ‘ever’ compared to ‘never’ product use. All models were run separately for each exposure and are not mutually adjusted for each other

^a^All models are adjusted for age at interview (continuous), cohort (NY-NCPP or NY-MBCP), and BMI (continuous)

^b^All models are adjusted for age at interview (continuous) and BMI (continuous)

After adjusting for confounders, there were no associations between duration of hair oil use in childhood and age at menarche (RR 1.0, 95% CI 0.8, 1.3) or between the duration of hair oil use in adulthood and mammographic breast density (percent density β 0.02, 95% CI -0.01, 0.05; dense area β 0.02, 95% CI -0.01, 0.05).

## Discussion

In the present study, we found that childhood hair product use was associated with earlier age at menarche, which is consistent with the existing literature [16, 19, 20]. Tiwary and colleagues were among the first to observe the use of topical applications of hair products during childhood in association with premature pubertal development (i.e., breast and pubic hair development) in African American girls [20]. Similarly, in the *Greater New York Hair Products Study,* we found that compared to other racial/ethnic groups, African American girls’ use of hair oils and use of perms/relaxers were independently associated with earlier age at menarche [19]. The evidence for the association of childhood hair product use and earlier menarche is further strengthened by in vitro proliferation assays that have found a brand of oil hair lotion, commonly used among black women, was associated with higher estrogenic activity [28].

On the other hand, we did not find a strong association between childhood or adult hair product use and mammographic density, a strong risk factor for breast cancer risk [11–13]. However, these associations may be limited by small sample sizes. We are not aware of any other published studies of hair product use in relation to mammographic density, but our results are consistent with the growing literature assessing hair products and breast cancer risk [17, 28–30]. Specifically, two studies recently found an increase in the odds of breast cancer risk associated with hair dye use in adulthood. One recent study found a 23% increase in the odds of breast cancer risk associated with hair dye use in Finnish women (OR 1.2, 95% CI 1.1, 1.4) [30] and another study using the *Women’s Circle of Health* found that among black women, the use of dark hair dye shades was associated with a 50% increased odds of breast cancer (OR 1.5, 95% CI 1.2, 1.9) [18]. In contrast, a meta-analysis of 14 breast cancer studies, that included cohort and case-control studies, reported a null association between hair dye use and breast cancer (RR 1.06, 95% CI 0.95, 1.18) [29]. Moreover, our finding of no association between perm/relaxer and mammographic density are also corroborated by analyses within the *Black Women’s Health Study* [17]. However, within the *Women’s Circle of Health Study*, findings suggest that adult use of relaxers was associated with breast cancer in white women, but not black women. In white women, the regular use of relaxers in adulthood was associated with a 1.6-fold increased odds of breast cancer compared to those who were non-regular users of relaxers in adulthood [18]. Rosenberg and colleagues found among over 48,000 black women with approximately 266,000 person-years of follow-up and 574 incident cases, that there was no increased risk of breast cancer associated with perm/relaxer use [17]. Myers and colleagues’ in vitro proliferation assays also did not detect estrogenic activity in their tested brand of hair perm/relaxer [28]. While the null associations were consistent for relaxer use in black women in both studies, it may be worth noting the possibility that white women using relaxers may be using different types of products than black women, which may have different chemical ingredients, as such these findings may suggest differences in exposures to chemicals and not necessarily differences in biology. Given prior literature supporting an association between EDC exposure and breast cancer risk, and consistent associations with earlier age at menarche, these chemicals may increase breast cancer risk through a pathway other than breast density. Prior literature on hair products, mainly hair dyes, had focused on these products as carcinogens, without attention to potential endocrine disruption pathways.

Our study is not without limitations and results should be viewed cautiously. First, structural confounding may limit our findings, as the degree of overlap in hair product use and patterns of use by race/ethnicity is quite limited. Specifically, a significantly greater proportion of non-Hispanic black women reported use of hair products compared to women of other racial/ethnic groups, which could limit the interpretation and conclusion of the race/ethnicity-adjusted analyses. However, we present models with and without adjustment for race/ethnicity. Second, when examining associations between childhood hair product use and age at menarche in our study, there were very few girls who reached menarche before age 11, which resulted in imprecise estimates. However, we observed an inverse association between childhood hair product use and age at menarche when menarche was modeled as a continuous outcome. Furthermore, in our previous study from the Greater New York Hair Products Study, which used the same cut point for reaching early age at menarche (<11 years), we also found an association between childhood hair oil use and age at menarche (relative risk 5.3, 95% CI 1.5, 19.1) [19]. Third, we had limited power to investigate associations for subgroups of hair products, resulting in the need to collapse several types of hair products into a single ‘other’ category. This category, along with hair oils and ever use of hair products was heterogeneous with respect to composition, which may have led to non-differential misclassification of exposure and bias toward the null. Nonetheless, we found significant associations between childhood hair oil use and age at menarche and suggestions of adult hair dye use and mammographic density. Fourth, unlike prior studies [15, 28], we did not examine the association between hair product brands and mammographic breast density. However, the ingredients for hair products varies over time [31] and disclosure of the exact ingredients is not required [32]. In addition, the current trend in personal care products, including hair products, is to be more organic and natural and many products which in the past contained common EDCs now advertise being “paraben and phthalate free” [31]. Therefore, a product brand analysis within our data may not inform current product ingredients. It will be important to investigate whether changes in these products over time reduce EDC exposure and the impact on health outcomes. Other challenges for hair product brand analyses are that the recall of brands used in childhood may be difficult and prone to error and there are likely to be different brands used across racial/ethnic groups. Fifth, we did not have the data available to adjust for childhood obesity, which might be an important factor to adjust for in our analyses as it is a predictor of the outcome. However, we believe childhood obesity could be a potential mediator of the association between hair product use and earlier age at menarche, as some of the chemicals in these products could be obesogenic. Lastly, age at menarche was reported retrospectively; however, studies suggest that retrospective reporting of age at menarche is moderately to highly reliable [33, 34].

Our study also has several strengths. We investigated these important associations in a multi-ethnic population, and our data on hair product exposure was measured using a questionnaire that has been used/validated in previous studies in racially/ethnically diverse populations. We also had data on subtypes of hair products and duration of exposure, and data on timing of exposure, which enabled us to investigate multiple critical and sensitive periods, which are particularly important in breast cancer risk.

Under the Federal Food, Drug, and Cosmetic Act, cosmetic products and ingredients, inclusive of hair products, do not need approval by the Food and Drug Administration (FDA) before they are marketed to the consumer [35]. The FDA does not impose regulations unless evidence suggests harm to human health or the environment [35] and the FDA at this time does not consider parabens or other EDCs often added to hair products to be associated with breast cancer risk [35]. However, research on associations between parabens and other EDCs and breast cancer, particularly in population groups who are long-term consumers of these products, is sparse. Given that black women are found to be the most frequent users of hair products [15–17], experience earlier pubertal development [36–38], and more aggressive breast cancer [39], larger mixed methods studies are needed to examine hair care product use and breast cancer risk. Studies need to incorporate personal interviews to detail product use and incorporate biological assessment of EDC concentrations (such as the inclusion of urine or blood biomarker data) to inform actual EDC exposure. Given the increase incidence in early onset breast cancer as well as the converging incidence trends between black and white women [2], further large-scale studies of early life exposures, including hair produce use in childhood are an essential next step to understanding changes in breast tissue characteristics across puberty and in midlife [40].

## Conclusions

In conclusion, our evidence and others suggest that childhood hair product use is associated with earlier age at menarche, an established risk factor for breast cancer. Moreover, we found that after adjusting for self-identified non-white race/ethnicity, hair product use remained associated with menarche; with relatively low power, we still observed borderline statistical significance for childhood hair oil use. We did not find an association between childhood or adult hair product use and mammographic breast density. Reducing use of hair products during childhood could potentially decrease EDC exposure with implications for earlier age at menarche and subsequent risk of breast cancer.
